# An Infectious Topic in Reticulate Evolution: Introgression and Hybridization in Animal Parasites

**DOI:** 10.3390/genes1010102

**Published:** 2010-06-09

**Authors:** Jillian T. Detwiler, Charles D. Criscione

**Affiliations:** Department of Biology, Texas A&M University, 3258 TAMU, College Station, TX 77843, USA; E-Mail: ccriscione@mail.bio.tamu.edu

**Keywords:** hybridization, introgression, parasites, helminths, protozoans

## Abstract

Little attention has been given to the role that introgression and hybridization have played in the evolution of parasites. Most studies are host-centric and ask if the hybrid of a free-living species is more or less susceptible to parasite infection. Here we focus on what is known about how introgression and hybridization have influenced the evolution of protozoan and helminth parasites of animals. There are reports of genome or gene introgression from distantly related taxa into apicomplexans and filarial nematodes. Most common are genetic based reports of potential hybridization among congeneric taxa, but in several cases, more work is needed to definitively conclude current hybridization. In the medically important *Trypanosoma* it is clear that some clonal lineages are the product of past hybridization events. Similarly, strong evidence exists for current hybridization in human helminths such as *Schistosoma* and *Ascaris*. There remain topics that warrant further examination such as the potential hybrid origin of polyploid platyhelminths. Furthermore, little work has investigated the phenotype or fitness, and even less the epidemiological significance of hybrid parasites.

## 1. Introduction

Reticulate genetic processes such as allele, gene, or genome (endosymbiont capture) introgression can have profound impacts on the ecological/evolutionary dynamics of populations and species. For example, hybridization between species or diverged populations could result in the transfer of adaptive traits, promote divergence via reinforcement (*i.e.*, selection for reproductive isolating mechanisms) when hybrids are less fit than parentals, lead to homogenization across the genomes of the interbreeding populations, or promote rapid adaptive diversification via the formation of hybrid species [1,2,3]. In relation to host-parasite interactions, such reticulate dynamics are of particular interest because host or parasite hybridization may impact host resistance/susceptibility or parasite infectivity, virulence, transmission, or host specificity.

The first major synthesis of the role of hybridization in host-parasite interactions was presented in a review by Fritz *et al.* [4], and an update was given in Wolinska *et al.* [5]. These reviews are important in drawing attention to the influence of reticulate dynamics on host-parasite interactions. However, the field, as reflected by the latter reviews, has largely taken a host-centric view such that the question of interest is whether the hybrid of a free-living species is more or less susceptible to parasite infection. In this regard, parasites are viewed as a selective force influencing the outcomes of reticulate evolution in the hosts. Here, we recognize that parasites themselves are subject to reticulate evolutionary dynamics. Thus, the aim of our review is to synthesize the current state of knowledge about the role of hybridization and introgression in the ecology and evolution of parasites. We restrict our review to protozoan and helminth (e.g., nematodes, platyhelminths) parasites of animals, but occasionally draw on other systems to illustrate concepts that are not adequately explored among animal parasites. In order to highlight some of the original concepts regarding hybridization and host-parasite interactions, we start with a brief synopsis of host hybridization. We then discuss lateral gene/genome transfer among distantly related taxa and parasites. In the latter portion of the review, we focus on the evidence for hybridization among closely related parasite species, diverged populations, or clonal lines and then highlight the ecological significance or phenotypic characteristics of parasite hybrids.

## 2. A brief synopsis of host hybridization

Fritz *et al.* [4] outlined five infection outcomes for a host hybrid relative to the parental lines (see also Box 2 of [5]): 1) additive, hybrid resistance is the average of the parental taxa; 2) dominance, hybrid resistance is more similar to either resistant or susceptible parental taxon; 3) hybrid resistance, hybrids are more resistant than either parental taxa; 4) hybrid susceptibility, hybrids are less resistant than either parental taxa; and 5) no difference, response of parents and hybrids is the same. In summarizing the results of 86 plant or animal studies (47 in Fritz [4] and 39 additional in Wolinska [5]), Wolinska *et al.* [5] state “Overall, these studies showed no clear trend in parasite responses to hybrids; depending on the host and parasite in question, different systems supported different infection scenarios. Moreover, the patterns obtained were often inconsistent in time and space.” Wolinska *et al.* [5] highlight that both variation in the genetic basis of resistance and context dependent environmental factors are often invoked to explain the variable patterns. They go on to postulate that frequency-dependent selection may be generating coevolutionary oscillations (Red Queen dynamics) such that parasites adapt to the common host genotype (*i.e.*, the hybrid genotype or parental genotypes). Therefore, a study that examines only a single time period may observe any of the above mentioned outcomes.

While Red Queen dynamics are plausible, there is another potential explanation for variable infection outcomes that has gone virtually unexplored. Parental host species may each have distinct genetic populations of parasites [4]. Infection outcomes in experiments may differ depending upon which host-associated parasite population was examined in the course of a study. Furthermore, if host hybridization leads to favorable conditions for parasite hybridization then there will be a genetically heterogeneous mixture of infectious propagules. Sloan *et al.* [6] showed that hybrids among different host-specific, anther-smut fungi had variable, but lower infection rates than the parental parasite genotypes on their hosts of origin. The point being that cryptic parasite genetic diversity or hybridization over space or time may lead to variable infection patterns among host parentals and hybrids. Among animal parasites, we are aware of only one study that has examined parasite population genetics across a host hybrid zone [7]. In this study, there was no phylogenetic structure of a nematode parasite among the hybrid and pure parental populations of two shrew species. We suggest that future studies examining host-hybrid fitness should begin to account for potential cryptic parasite genetic structure.

Studies on animal hybrids are going beyond questions of hybrid fitness in relation to parasitism and are now asking what introgressed genes may be playing a role in parasite susceptibility or resistance. For example, Slotman *et al.* [8] and Parmakelis *et al.* [9] used molecular evolutionary analyses to examine mosquito loci (within the *Anopheles gambiae* complex) implicated in the resistance of malaria infections. Analyses at several loci have indicated introgression of alleles within this host species complex. At one locus (*LRIM1*, a leucine-rich repeat immune protein that is an important malaria antagonist), there was evidence for adaptive evolution and introgression from *An. arabiensis* into *An. gambiae*. Recognizing host loci involved in reticulate dynamics will aid in our understanding of the spatial and host use distributions of parasites.

## 3. Gene/genome introgression from distantly related taxa into parasites

There is accumulating evidence that animal parasites have acquired genes or endosymbiotic genomes from other organisms. Genes from plants, algae, cyanobacteria, and eubacteria have been transferred either by intracellular gene transfer from an endosymbiont or horizontal gene transfer [10]. Phylogenetic methods, along with examinations of codon bias, uneven distributions of genes, and BLAST searches are commonly used to identify sources of transfer events [11]. For example, the protozoan parasite *Cryptosporidium parvum* forms a monophyletic clade with a green alga instead of other apicomplexans in the gene phylogeny of uridine kinase-uracil phosphoribosyltransferase [12]. In animal parasites, most lateral gene transfers have been identified in human parasites in the groups Nematoda, Apicomplexa and Kinetoplastida. In part, this research bias is due to the genomic resources available for a few species in these groups. One of the major motivations for examining gene transfer in parasitic organisms is to identify genes that are of bacterial origin. Because such genes are less likely to have eukaryotic homology, they may be good targets for drug treatment therapies [13,14].

In most filarial nematodes (Onchocercidae), a mutualistic interaction appears to have evolved with the obligate intracellular bacteria *Wolbachia* [15]. Normal development and fertility of the nematode parasite is linked to *Wolbachia* infections [16,17]. Furthermore, some *Wolbachia* strains in filarial nematodes have undergone genome reduction. Compared to the *Wolbachia* genome from *Drosophila melanogaster*, the *Wolbachia* genome from the filarial nematode *Brugia malayi* is smaller [18]. Gene loss appears to have occurred in genes associated with cell wall biogenesis, which may reflect the mutualistic relationship of their interaction. Conversely, *Wolbachia* maintain genes associated with metabolic functions such as riboflavin and heme synthesis, which are absent in their host nematode’s genome [18]. Evidence of gene transfer is found in species with current *Wolbachia* infections [15,19]. These sequences appear as degenerate fragments in several filarial parasite species’ genomes [19]. Other filarial parasite species are *Wolbachia*-free, yet also contain genes with high homology to *Wolbachia* [20,21]. It may be that lateral gene transfer incorporated key genes into the filarial worm nuclear genome, allowing the nematode to live without its former endosymbiont. There are no reports of nematode DNA being incorporated into the *Wolbachia* genome [18].

Protozoan parasites contain genes from several different sources. For example, most parasitic apicomplexans such as *Plasmodium* possess an apicoplast organelle that is hypothesized to be an ancient secondary endosymbiosis with an algal cell (exceptions include *Cryptosporidium* and *Gregarina*) [14]. Even though *Cryptosporidium parvum* lacks an apicoplast, plant-like genes were detected in its genome [10]. Huang *et al.* [10] also hypothesized that genes from algal nuclear and chloroplast (cyanobacteria) were introduced via intracellular gene transfer from an endosymbiont, while proteobacteria genes were horizontally transferred. After an intracellular transfer from a red algae endosymbiont, the chloroplast and mitochondria were lost, and the plastid genome (apicoplast) in apicomplexans was reduced [23]. Unique organelles, like the glycosome in kinetoplastids (*Trypanosoma* and *Leishmania*) contain many plant-like genes likely transferred by endosymbiosis in a single event [24]. As in the *Wolbachia*-filarial system, transferred genes can perform essential functions for the protozoan parasite. For example, current evidence indicates that in *Cryptosporidium parvum,* the genes involved in nucleotide biosynthesis originated via eubacterial horizontal gene transfer [12,25]. Likewise, horizontally transferred genes encode important enzymes for fermentation and other functions that are essential to the anaerobic lifestyle of *Giardia lamblia* [26,27].

Taxon sampling is the most problematic aspect of detecting horizontal gene transfer in animal parasites. Phylogenetic analysis with inadequate taxonomic diversity may suggest a transfer event; however, the addition of more taxa could change the conclusion to shared common ancestry [28]. Genomic resources for non-model animal parasites will help fill these phylogenetic gaps. As genomic resources are generated for a wider array of parasite taxa, researchers will need to conduct adequate searches of databases. Misleading reports of gene transfer exist because particular databases were not investigated [28]. Concurrently, the number of platforms to analyze large sets of phylogenetic data is also increasing [11,29]. 

## 4. Evidence of hybridization among closely related parasite taxa.

**Figure 1:**
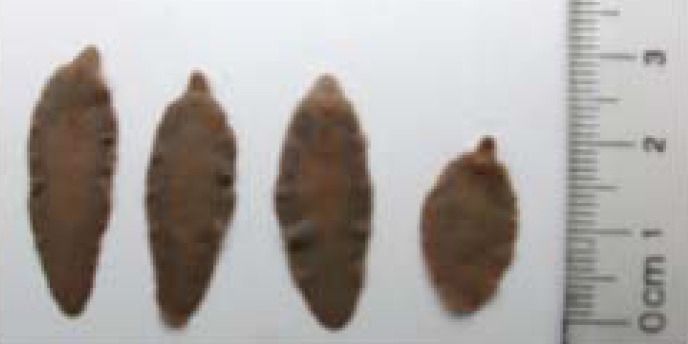
Fasciola specimens collected from goats in a province (Yen Bai, YB) of North Vietnam. The first three flukes (left to right) show the normal shape of *Fasciola gigantica* while the last individual resembles both the hybrid and *F. hepatica*. The figure shows that hybrids can look like one of the parents, thus illustrating the point that it is difficult to use parasite morphology to identify hybrids. Reprinted from Thanh *et al.* [32] with permission from Elsevier.

Our understanding of the frequency and ecological/evolutionary importance of parasite hybridization among closely related species or populations lags far behind that of free-living organisms [30]. In part, this lag exists because parasites have limited morphological characters or other diagnosable phenotypes. Thus, there are inherent difficulties in identifying possible intermediate phenotypes that would be characteristic of a hybrid (e.g., Figure 1). While morphology has been used to speculate on the presence of parasite hybrids (e.g., *Fasciola*, [31,32], *Schistosoma* [33], *Gyrodactylus* [34,35]), genetic markers, from protein electrophoretic data to sequence data, have greatly enhanced our ability to detect hybrids. In Table 1, we summarize studies that provide evidence for hybridization in nature. Table 1 is comprehensive only in terms of the different taxonomic groups showing hybridization rather than reporting each individual study as many of the more recent papers often reference the history of hybridization in their respective organisms. Below we highlight how hybrids have been identified and discuss some of the better supported examples.

### 4.1. Helminths

For most parasitic helminths, hybridization has been described between recognized congeneric species or host-associated populations. Table 1 lists four digenean genera (*Schistosoma*, *Fasciola,* *Paragonimus,* and *Diplostomum*), two cestode (*Taenia* and *Echinococcus*), one monogenean (*Gyrodactylus*), and five nematode (*Ascaris, Contracaecum, Parascaris, Pseudoterranova,* and *Paramacropostrongylus*) where there is support for current (within 2 generations) or historical hybridization. A common approach to identifying hybrids is the use of parental taxa specific markers (PT-SM in Table 1). This approach is seen in earlier studies that used protein electrophoretic data to detect species-specific alleles at nuclear loci. For example, isoenzymes at two loci displayed distinctive banding patterns in *Schistosoma haematobium* and *S. mattheei* [36]. Mixed (heterozygote) banding patterns were identified from individual parasite offspring collected from humans, thus indicating natural hybridization [36]. This approach assumes the alleles are fixed in the parental species. Thus, one would need adequate sample sizes in “pure” parental populations to confirm marker fixation. However, if “pure” populations are geographically separate from the hybrid population of interest, the assumption of fixation is confounded with geography (*i.e.*, allele frequencies may vary due to geography and not species status). Nuclear-mitochondrial discordance is also commonly used to identify putative hybrids (Table 1). For instance, Okamoto *et al.* [37] found individuals with mitochondrial DNA of *Taenia saginata*, but in a nuclear background of *T. asiatica*. The caveat of nuclear-mitochondrial discordance is that these data alone cannot tease apart incomplete lineage sorting, historical introgression, or contemporary hybridization. More recently, studies [38,39] (discussed below) have utilized highly polymorphic markers such as microsatellites and model-based Bayesian clustering methods [40,41,42]. These methods are advantageous because analyses can be conducted when no taxa-specific markers exist, and pure samples of parental taxa are not required [40]. Furthermore, the results from Bayesian clustering analyses indicate contemporary hybridization as these methods only detect hybrids going back approximately two to three generations [40].

**Table 1:** Studies providing evidence for natural hybridization in animal parasites. Types of data used to infer hybridization include: experimental infections (EI), parental taxa specific markers (PT-SM), nuclear-mitochondrial discordance (NMD), model-based Bayesian clustering inference (BC). We only included EI-based studies if there was also evidence of hybridization in nature stemming from PT-SM, NMD, or BC data.

Hybridizing taxa	EI	PT-SM	NMD	BC
Human and pig associated *Ascaris* populations (Nematoda)		88	89	39
*Contracaecum* populations I and II (Nematoda)		90		
*Paramacropostrongylus ingalis* and *P. typicus* (Nematoda)		91		
*Parascaris univalense* and *P. equorum* (Nematoda)		92		
*Pseudoterranova decipiens* sp. A and B (Nematoda)		93		
Host-associated *Gyrodactylus salaris* populations (Platyhelminthes)		77, 94		
*Diplostomum* cryptic species (Platyhelminthes)		82	82	
*Fasciola hepatica* and *F. gigantica* (Platyhelminthes)		95, 96	32, 97-99	
*Paragonimus westermani* species complex (Platyhelminthes)		100, 101	102	
*Schistosoma mattheei* and *S. haematobium* (Platyhelminthes)	36	36, 103		
*Schistosoma bovis* and *S. curassoni* (Platyhelminthes)	33	33		
*Schistosoma bovis* and *S. haematobium* (Platyhelminthes)	104		105	
*Schistosoma haematobium and S. guineensis* (Platyhelminthes)	74,75	73,74,75		
*Schistosoma mansoni* and *S. rodhaini* (Platyhelminthes)	71, 104, 106		38, 45	38
Strains of *Echinococcus granulosus* (Platyhelminthes)			107	
*Taenia saginata* and *T. asiatica* (Platyhelminthes)			37	
*Leishmania braziliensis* and *L. peruviana* in subgenus *Viannia* (Euglenozoa)	67	53, 55		
*Leishmania braziliensis* and *L. panamensis* in subgenus *Viannia* (Euglenozoa)		108		
*Leishmania naiffi* and *L. lainsoni* in subgenus *Viannia* (Euglenozoa)		109		
*Leishmania infantum* strains in subgenus *Leishmania* (Euglenozoa)		110, 111		110, 111
*Leishmania donovani* strains in subgenus *Leishmania* (Euglenozoa)		112		
*Leishmania infantum* and *L. major* in subgenus *Leishmania* (Euglenozoa)	68	54		
*Trypanosoma cruzi* discrete typing units (DTUs)/lineages	60	52, 57-59, 112-115	113	

Among helminths, schistosomes (blood flukes) are the most comprehensively studied and provide the best evidence of hybridization from many forms of data. For illustration we discuss *S. mansoni-rodhaini* hybridization, but refer readers to Table 1 and reviews in [43,44] for additional schistosome examples. *Schistosoma rodhaini* is predominately a parasite of rodents and *S. mansoni* of humans; however, both parasites have been reported from humans and rodents, respectively (reviewed in [45]). Since the 1950’s several laboratory crosses have demonstrated that viable offspring could be generated (Table 1). Yet, the first evidence of a natural hybrid, which was based on nuclear-mitochondrial discordance, was not reported until 2003 [45]. Recent work using microsatellites and Bayesian clustering has confirmed that hybridization is a contemporary phenomenon between *S. mansoni* and *S. rodhaini* [38]. Furthermore, introgression appears asymmetric, going from *S. rodhaini* to *S. mansoni*. To our knowledge, the only other helminth example of contemporary hybridization is between human and pig associated populations of roundworms (traditionally referred to as *Ascaris lumbricoides* and *A. suum*, respectively). Bayesian clustering methods and microsatellites revealed evidence for hybridization in sympatric populations within both Guatemala and China. These results indicate that there must be contemporary interbreeding and thus, necessarily recent cross transmission among host species [39]. Evidence for recent introgression in *Ascaris* and *Schistosoma* raises important issues with regards to the epidemiology of these parasites such as the potential for cross-transmission between humans and reservoir hosts (as hybridization indicates adults of the two parental taxa must be coinfecting the same final hosts) and the potential for the transfer of adaptive alleles across species/host-associated boundaries.

It is noteworthy to mention that polyploids with apparent asexual reproduction have been reported in several parasitic platyhelminths [31,46,47]. Several studies on *Fasciola* and *Paragonimus* have presented data that suggest polyploids are of hybrid origin (*i.e.*, allopolyploids) (Table 1). For example in Vietnam, Itagaki *et al.* [48] found that 18/22 triploid *Fasciola* specimens had a heterozygous genotype at the internal transcribed spacer 1 region of the rDNA genotype where one sequence was of the *F. hepatica* type and the other of *F. gigantica*. The mitochondrial type was of *F. gigantica*, but of possible Japanese or Korean origin, thus suggesting the hybrid was introduced rather than originating within Vietnam. In contrast, the hybrid origin of triploid or tetraploid *Paragonimus westermani* is still being debated where some evidence suggests hybridization among different populations/species whereas other data support an autopolyploid origin in this hermaphroditic species (see [49,50]). Regardless of the origin of the polyploids, the generation of asexual lineages presents another interesting reticulate evolutionary dynamic, *i.e.*, the potential introgression between asexual lineages or between asexual and sexual. For instance, in the sperm-dependent parthenogenetic triploids of the free-living flatworm *Schmidtea polychroa*, occasional sex has been documented [51]. Similar reticulate dynamics may be occurring in *P. westermani* as penetration of haploid sperm from diploids into unreduced eggs of triploids has been observed and population genetic data indicate that the alleles in tetraploids are present in sympatric diploids and triploids (reviewed in [50]).

### 4.2. Protozoans

Whereas studies on helminth species largely focus on hybridization among recognized congeneric species, data on protozoan parasites often reflect recombined clonal lineages (Table 1). Historically, protozoan taxa were thought to reproduce clonally and therefore hybridization/genetic exchange was thought to play a minimal role in their evolution [52]. However, it is now evident that recombination has played and/or currently plays a significant role in the evolution of parasitic protozoans (Table 1). Several studies indicate that hybridization occurs between *Leishmania* species. In *Trypanosoma*, hybridization is reported to occur within the *T. cruzi* and *T. brucei* species complexes. Likewise, a few studies suggest potential hybridization in the *Giardia duodenalis* species complex (Table 1).

In *Leishmania*, natural hybridization has been detected between species within, but not between the subgenera *Leishmania* and *Viannia.* Within a subgenus, hybridization can occur between closely related species that are considered to be in the same species complex [53] or between species from different species complexes [54]. The parental taxa specific markers method (or clonal lineage-specific) with either multilocus enzyme electrophoresis or sequence data is commonly used to characterize parental taxa and their putative hybrids (Table 1). This method is greatly enhanced and provides strong evidence for genetic exchange when a genome-wide approach is used. For example, using 19 loci across at least six chromosomes, hybrids between *Leishmania infantum* and *L. major* were identified because at all loci, these samples were heterozygous for alleles that were fixed in parental taxa [54]. In several epidemiological studies, a low frequency of isolates that are regarded as hybrids is often reported (Table 1). However, a recent study conducted in a local contact zone observed just as many hosts (including humans and dogs) infected with hybrids of *Leishmania braziliensis*/*L. peruviana* as infected with the parent *L. braziliensis* [55]. Nearly half of the hybrids (46%, 12/26) were of a single multilocus genotype, but the presence of other hybrid genotypes suggests that several hybridization or backcrossing events may have occurred in this area. Both dogs and humans were infected with the common hybrid genotype, which raises concerns that dogs could act as reservoir hosts for emergent parasite hybrids. It should be noted that in some cases putative hybrids cannot be differentiated from mixed infections. This is particularly relevant because hosts may accumulate infections from different genotypes, which can persist for long periods of time in the host [54]. Verifying hybrid status from natural isolates requires confirmation by cloning isolates and retyping several individuals from each clonal type [54].

**Figure 2:**
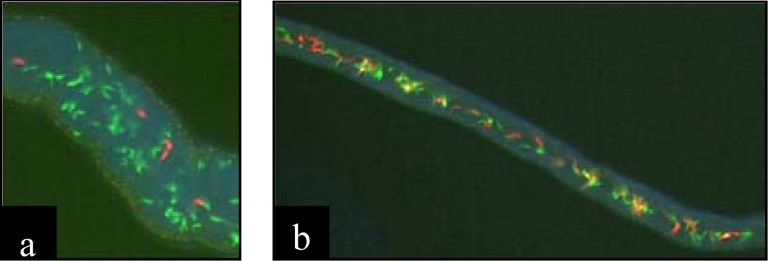
Demonstrating that fluorescence microscopy can identify trypanosome hybrids. Fluorescent proteins indicate the hybrid (yellow) or parental status (red and green) of trypanosome parasites. **(a)** Close proximity of red and green parental trypanosomes in salivary glands at early establishment. Flies dissected at 20 days. **(b)** Yellow hybrids with red and green parental trypanosomes in salivary glands. Flies dissected at 27 days. Trypanosomes are 20–30 μm in length. Reprinted from Gibson *et al.* [63], originally published by BioMed Central.

Among trypanosomes, two species complexes offer independent evidence that hybridization plays a role in the evolution of this group. *Trypanosoma cruzi* is a species complex consisting of six DTUs (discrete typing units) that are each recognized by characteristic multilocus genotypes and strong linkage disequilibrium [56]. There is strong evidence from a variety of molecular approaches and markers for two historical hybridization events within the *T. cruzi* complex (Table 1). Four of the six major DTUs are likely of hybrid origin because across many loci, they have fixed heterozygosity for alleles that are fixed in the parental DTUs [57,58,59]. Laboratory crosses have not been able to demonstrate inter-DTU hybridization, but they have confirmed that genetic exchange can occur between lineages within a DTU (Gaunt *et al.*, 2003). In contrast, genetic exchange among *T. brucei* strains and subspecies *T. b. rhodesiense* and *T. b. gambiense* was demonstrated in the laboratory (see Table 1 in [61]). Some crosses result in progeny that do not fit Mendelian expectations, therefore suggesting that selection with the salivary glands of the tsetse fly may be occurring [62]. Recent developments with fluorescence microscopy have confirmed that genetic exchange is exclusive to the salivary glands [63]. *Trypanosoma brucei* parents were transfected with either a red or green fluorescent protein, and due to co-expression the hybrids appeared as yellow (Figure 2). Overall, it is critical to understand the frequency and ecological niches of the DTUs, especially because recombinant parasites have been associated with both severe acute and chronic Chagas in humans throughout South America [56].

*Giardia duodenalis* (syn *G. lamblia*) is another protozoan that was thought to be strictly clonal [64]. This parasite is a globally distributed parasite characterized into seven highly divergent genetic assemblages [65]. Genetic variation within each assemblage can be very different. Several allozyme and isozyme studies demonstrated that Assemblage A has fixed homozygosity, whereas assemblage B contains some heterozygotes [64]. Nessilquist *et al.* [64] suggested that both gene transfer and recombination may explain some of the observed heterozygosity in particular assemblages. For example, gene transfer was suggested when alleles fixed in A were found in B assemblage isolates. Recombination was detected with at least three different statistical tests on sequence data. Although evidence varied among loci, recombination was detected within and between assemblages. Thus, it appears that like *Trypanosoma* and *Leishmania,* the *G. duodenalis* species complex occasionally undergoes hybridization, which creates recombinant lineages.

## 5. Ecological and evolutionary significance of parasite hybrids. 

Hybrids are new genetic variants and thus, may have novel phenotypes that promote ecological diversification [30]. For instance, relative to their parents, plant-pathogen hybrids can colonize new host species, have increased or decreased virulence, and even exhibit a new mode of reproduction (sexual *vs.* asexual) [66]. In the above section, we focused on the occurrence of hybridization. Here, we discuss studies that investigate the ecological/evolutionary significance of parasite hybrids. In animal parasites, hybrids have been investigated for changes in infectivity as defined by infection intensity or density within hosts, or infection prevalence. Other hybrid traits that have been examined include larval emergence behavior, and hybrid reproductive output or viability. Also of interest is the mating behavior of parental taxa that may lead to the genetic assimilation of one of the parental forms. To facilitate discussion on the ecological and evolutionary significance of parasite hybridization, we adopt Fritz *et al.*’s [4] host-hybrid infection scenarios for parasites. Thus, the phenotype of a parasite hybrid may be 1) additive, hybrid phenotype is the average between two parental taxa; 2) dominance, hybrid phenotype is more similar to either parent; 3) increased, hybrid phenotype exceeds either parent; 4) reduced, hybrid phenotype is less than either parent; 5) no difference, hybrids and parents have the same phenotype.

**Figure 3:**
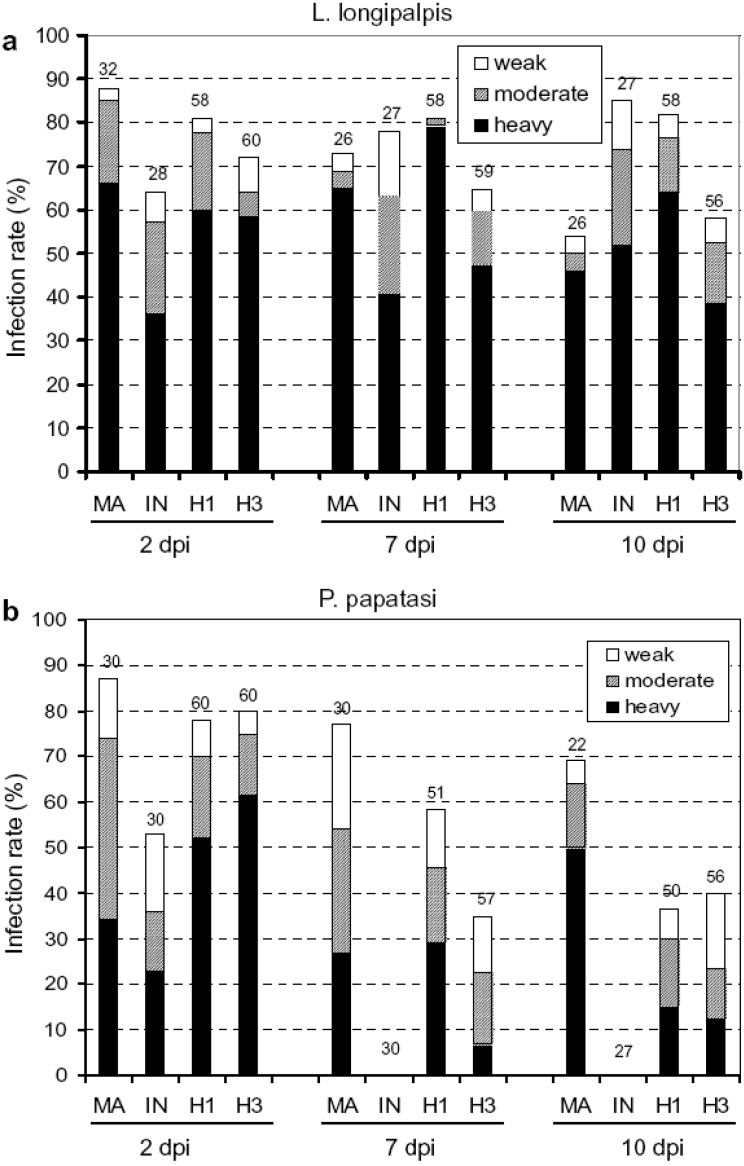
Development of *Leishmania* hybrids in *Lutzomyia longipalpis* and *Phlebotomus paptasi*. Infection rates and density of *Leishmania major* (MA), *Leishmania infantum* (IN), hybrid LEM4891 (H1) and hybrid LEM4833 (H3) in sand fly midgut on days 2, 7 and 10 p.i. Infections were classified into three categories: heavy (more than 1000 promastigotes per gut) – black bars, moderate (100-1000) – grey bars, light (1-100) – white bars. Numbers above the bars indicate the number of dissected females. **(a)** Development in *L. longipalpis*: the infection rate and the intensity of infection did not differ between *Leishmania* strains studied. **(b)** Development in *P. papatasi*: on days 7 and 10 p.i., the infection rate and the intensity of infection significantly differed between *L. major*, hybrids and *L. infantum*. Reprinted from Volf *et al.* [68] with permission from Elsevier.

Host susceptibility/resistance is the most common trait examined with regards to hybrid host-parasite interactions [4,5]. However, the parasite centric complement of host susceptibility, *i.e.*, hybrid parasite infectivity, has only been assessed in a few animal parasite systems. *In vitro* experiments with five different strains of *L. braziliensis*, *L. peruviana*, and their hybrid indicated that promastigote growth rate and infectivity (density) exhibited the dominance scenario because hybrids were no different from *L. peruviana*, but grew significantly slower and were less dense than *L. braziliensis* promastigotes [67]. Volf *et al.* [68] experimentally examined infectivity of *Leishmania major*-*L. infantum* hybrids (two strains obtained from HIV patients) in two insect vectors, *Phlebotomus papatasi* and *Lutzomyia* *longipalpis*. The former vector supports development for *L. major*, but no other *Leishmania* species, whereas *L.* *longipalpis* can support the development of a broad range of *Leishmania* species. In *L.* *longipalpis*, there was no difference between the hybrid and parental strains early in the infection. At a later stage of infection one hybrid line had a lower prevalence similar to that found in *L. major,* and the other hybrid line had higher prevalence as found in *L. infantum* (Figure 3). Thus, there was a shift from no difference to the dominance category later in the infection. In contrast, hybrid infectivity was additive for both hybrid strains (35-38% prevalence) in *P. papatasi* where *L. major* had ~69% infection rate and *L. infantum* had 0% (Figure 3). A major surface molecule, lipophosphoglycan (LPG), is critical for *L. major* to establish infection in *P. papatasi* [69]. Volf *et al.* [68] found that both hybrid strains expressed *L. major* LPG. Immunofluorescence intensity was intermediate in hybrids and absent in *L. infantum.* These findings have important epidemiological implications as *P. papatasi* has a widespread range in Europe, Africa, and Asia and thus, there exists a potential to spread a new hybrid parasite over a vast area [68]. Furthermore, these results emphasize how the phenotypes of parasite hybrids may vary in different host species backgrounds.

Variation in infectivity among different host backgrounds was also demonstrated in reciprocal infection experiments of sympatric/allopatric populations of *Microphallus* trematodes and their snail hosts [70]. Infectivity of F1 hybrid parasites (generated by crossing two allopatric populations of parasites) was lower than the average infectivity of parentals with sympatric hosts. However, there was no difference between the hybrids and the parentals with allopatric hosts (*i.e.*, host populations where neither parental parasite originated). The results did not support additive or complete dominance scenarios. Dybdahl *et al.* [70] propose that locally adapted gene-complexes were disrupted by hybridization, thus leading to lower fitness (*i.e.*, outbreeding depression) in the hybrids.

The timing of larval (cercarial) emergence from snails has been investigated in schistosome hybrids of *S. mansoni* and *S. rodhaini*. This trait is epidemiologically important as it may promote the transmission of the parasite to the suitable mammalian definitive host. Théron *et al.* [71] set up two crosses: a late afternoon emergence *S. mansoni* with evening emergence *S. rodhaini* and a mid-day emergence *S. mansoni* with evening emergence *S. rodhaini.* The former cross produced two emergence peaks, but with most cercariae emerging in the evening. In contrast, the latter cross produced two peaks, but with most cercariae emerging mid-day; the F2 of this cross had the same pattern. At first glance, the F1 phenotypes appear to display a dominant phenotype. However the F2 was identical to the F1, thus the underlying genetic mechanism to the emergence phenotype is unclear. From field-collected parasites, Steinauer *et al.* [38] found that individuals with hybrid ancestry largely had an emergence time similar to pure *S. mansoni* individuals. One individual did display two peaks of emergence that coincided with the two parental times. However, the authors caution that small sample size and possible mixed ancestry of hybrid classes preclude definitive conclusions. 

Reproductive output was examined in hybrids of schistosome and echinostome trematodes. Laboratory F1 hybrids of *S. haematobium* and *S. mattheei* produced more eggs than either of the parents in hamsters [36]. In addition, the hybrids exhibited increased infectivity in both snail and hamster hosts and increased growth and maturation rate compared to the parent species. Echinostome hybrids were created by crossing two different populations of *E. caproni* originating from Egypt and Madagascar [72]. The fecundity (egg output) of F1 hybrids was similar to that of the mid-parent, but the fecundity of the F2 and F3 parasites was significantly lower than that of F1 and that of the mid-parent. Thus, the authors suggest hybrid breakdown as a possible reproductive isolating mechanism in echinostomes. Hybrid breakdown was also suggested by decreasing egg size from the F1 to F4 generation of a *Schistosoma curassoni* and *S. bovis* cross [33].

Besides the immediate phenotype of parasite hybrids, other important ecological and evolutionary consequences of parasite hybridization that have been addressed include the potential for genetic assimilation of one of the parental taxa and colonization of a new host species by a parasite hybrid. In some areas of Cameroon, Webster *et al.* [73] indicate that over a 25 year period *Schistosoma guineensis* has been replaced by *S. haematobium* via introgressive hybridization. Laboratory crosses suggest that mating dynamics between the parental species may be responsible for this outcome (reviewed in [43]). For example, males of *S. haematobium* were better at pairing with females of either species compared to males of *S. guineensis* [74]. Additional experiments revealed that F1 hybrids were able to take females from *S.* *guineensis* homospecific pairs more easily compared with females from *S. haematobium* homospecific pairs [75].

Parasite hybrids may have new phenotypes which allow them to colonize novel host species. Host switching has characterized the evolution of the parthenogenetic, but potentially hermaphroditic monogenes of the genus *Gyrodactylus* [76]. Recent data from a nuclear locus indicate that *G.* *salaris* on Baltic salmon has fixed heterozygosity for alleles that are fixed in two geographic strains of *G. salaris* parasitizing grayling fish [77]. Mitochondrial data show the Baltic salmon clade of *G. salaris* is monophyletic suggesting a monoclonal lineage of hybrid origin. Interestingly, Baltic salmon are only parasitized by the hybrids, whereas other sympatric fish species harbor several species of *Gyrodactylus* [78]. The authors propose that such hybridization events may provide one explanation and mechanism for host switching and speciation in the genus *Gyrodactylus*.

## 6. Concluding remarks and future directions

Data on reticulate evolutionary dynamics in animal parasites are still emerging. It is evident that hybridization and introgression have occurred at multiple levels (e.g., between closely related species to endosymbiont genome capture). Yet, the current state of knowledge does not provide a broad enough perspective to draw generalized conclusions about reticulate evolution in animal parasites. For instance, given the number of species and taxonomic diversity of animal parasites [79], the paucity of studies represented in Table 1 might lead one to conclude that hybridization is rare in animal parasites. However, this is likely a consequence of limited exploration rather than a reality. Thus, we highlight two main areas for future research.

First, more data are needed on the frequency and geographic patterns of hybridization in animal parasites. Most studies we have referenced in this review focus on parasites of humans or human-associated animals, thus investigation for hybrids in other animal parasite systems is warranted. An increasing number of studies in non-human systems are finding evidence of cryptic parasite species that may co-occur within the same final host [80,81], thus leading to the potential for matings between closely related species. For example, in a study aimed at identifying cryptic parasite diversity, Locke *et al.* [82] detected signatures of potential hybridization between cryptic species of *Diplostomum* trematodes. Among species groups (e.g., *Fasciola*, *Paragonimus*) where hybridization has been reported from multiple geographic localities, more data are needed to determine if hybrids are the result of single event and subsequent spread, or if multiple hybridization events have occurred. It is also interesting to speculate if humans have facilitated some of these parasite hybridization events (e.g., via habitat alterations or movement of hosts over vast geographic areas). Although we advocate additional exploration, we also highlight that many of the current reports have relied on limited data sets (e.g., few molecular markers and small sample size) to infer hybridization (Table 1). Thus, more data are needed to conclude current hybridization, especially if we are trying to assess the potential for ongoing gene exchange in important epidemiological traits such as drug resistance or host specificity.

The second avenue we suggest for future research is for a greater concentration on the ecological and evolutionary significance of hybrids. Major themes in parasite biology include infectivity, virulence, transmission, and host specificity, but not much is known about the potential influence of parasite hybridization in these areas. Indeed, little work has addressed the infectivity of parasite hybrids in different host species or host backgrounds [36,68,70]. Furthermore, hybrid parasite fitness relative to parentals has rarely been assessed [36,72], which we find surprising given this is what is commonly assessed in free-living hybrid organisms [66]. The increasing genomic resources for some human related parasites will enable more studies on parasite hybrid phenotypes and their underlying genetic control. The genome has been sequenced and a genome wide linkage map is available for *Schistosoma mansoni*, which can hybridize with *S. rodhaini* [83,84]. Similar resources are available for trypanosomes [85,86,87]. These resources will facilitate the examination of hybrid genome architecture and the mapping of traits that differ in the parental species. Thus, these tools will not only enable a better understanding of reticulate evolution in parasites, but also allow researchers to take advantage of natural reticulate dynamics to better understand important epidemiological traits.

As the hybridization literature develops in both animal parasites and free-living species, we can address broader evolutionary questions that compare the processes and consequences of hybridization in parasitic and free-living taxa. For example, are there certain life history traits such as life cycle patterns or levels of host specificity that predispose certain parasites to hybridization? Data from some free-living systems has shown that the transfer of alleles has allowed introgressed individuals to survive in the face of environmental stressors (e.g., iris hybrids under flooded conditions; Arnold this issue). Would we expect to find such patterns in parasites and what might be the stressors (host immune response?) and parasite genes involved (immune evasion or antigen expressing genes?). The answers to these questions await more data and it is our hope that this review helps promote parasite reticulate evolution as an infectious topic of study. 
